# Access to Top-Cited Emergency Care Articles (Published Between 2012 and 2016) Without Subscription

**DOI:** 10.5811/westjem.2019.2.40957

**Published:** 2019-04-16

**Authors:** Murad Al Hamzy, Dominique de Villiers, Megan Banner, Hein Lamprecht, Stevan R. Bruijns

**Affiliations:** * Stellenbosch University, Division of Emergency Medicine, Cape Town, South Africa; † University of Cape Town, Division of Emergency Medicine, Cape Town, South Africa

## Abstract

**Introduction:**

Unrestricted access to journal publications speeds research progress, productivity, and knowledge translation, which in turn develops and promotes the efficient dissemination of content. We describe access to the 500 most-cited emergency medicine (EM) articles (published between 2012 and 2016) in terms of publisher-based access (open access or subscription), alternate access routes (self-archived or author provided), and relative cost of access.

**Methods:**

We used the Scopus database to identify the 500 most-cited EM articles published between 2012 and 2016. Access status was collected from the journal publisher. For studies not available via open access, we searched on Google, Google Scholar, Researchgate, Academia.edu, and the Unpaywall and Open Access Button browser plugins to locate self-archived copies. We contacted corresponding authors of the remaining inaccessible studies for a copy of each of their articles. We collected article processing and access costs from the journal publishers, and then calculated relative cost differences using the World Bank purchasing power parity index for the United States (U.S.), Germany, Turkey, China, Brazil, South Africa, and Australia. This allows costs to be understood relative to the economic context of the countries from which they originated.

**Results:**

We identified 500 articles for inclusion in the study. Of these, 167 (33%) were published in an open access format. Of the remaining 333 (67%), 204 (61%) were available elsewhere on the internet, 18 (4%) were provided by the authors, and 111 (22%) were accessible by subscription only. The mean article processing and access charges were $2,518.62 and $44.78, respectively. These costs were 2.24, 1.75, 2.28 and 1.56 times more expensive for South African, Chinese, Turkish, and Brazilian authors, respectively, than for U.S. authors (p<0.001 all).

**Conclusion:**

Despite the advantage of open access publication for knowledge translation, social responsibility, and increased citation, one in five of the 500 EM articles were accessible only via subscription. Access for scientists from upper-middle income countries was significantly hampered by cost. It is important to acknowledge the value this has for authors from low- and middle-income countries. Authors should also consider the citation advantage afforded by open access publishing when deciding where to publish.

## INTRODUCTION

Access to key academic literature is vital for authors, scientists and clinicians, especially those working in low- and middle-income countries.^1^,^2^ Although open access publishing has made a large contribution to improved accessibility of research, article processing costs (the cost to publish open access) can be expensive for any author.^1^,^3^ Subscriptions and single-article access costs are also expensive, and as a result subscriptions are frequently delegated to academic libraries.^4^ However, limitations in journal subscriptions available at such libraries have resulted in scientists and clinicians having to pay article access fees, find an archived copy in an online repository, or contact the author to ask for a copy of his or her work.^5^ Access to published articles, and the options for publishing new work, are limited for authors, scientists and clinicians without academic library access. This problem disproportionately affects those from less developed settings,^5^ and is likely to affect the local knowledge economies.^6^

Unrestricted access to research improves research progress, productivity, and knowledge translation. These in turn develop and promote the efficient dissemination of content in an ever-expanding knowledge cycle.^4^ As a result, clinicians from different health institutions across the world are connected in the dissemination of new findings, and they have the information available to make the most appropriate decisions concerning patient care. Access to research literature is, therefore, an important part of disseminating emergency care information globally and locally. However, it is not known how accessible emergency care research is on a global level, nor what costs are involved. We describe access to the 500 most-cited emergency medicine (EM) articles (published between 2012 and 2016) in terms of publisher-based access (open access or subscription), alternate access routes (self-archived or author provided), and the relative cost of access (article access costs or article processing costs).

## METHODS

This was a retrospective, cross-sectional study using secondary, published data. We searched for articles via Scopus and SciVal (both Elsevier, Amsterdam) to identify the 500 most-cited EM articles published between 2012 and 2016. Scopus is the largest abstract and citation database of peer-reviewed literature. SciVal is a powerful data engine that can be used (amongst a vast number of other functions) to interrogate the Scopus database. We used it to perform an automated keyword search for EM articles, along with citation counts and journal, author and publisher details. Articles were then ranked using their citation count to allow selection of the sample.

Each of the included articles was manually checked to identify their open access status via the publisher’s websites. Where articles were not available open access from the publisher’s website (subscription-based articles), we used the article title to interrogate Google, Google Scholar (https://scholar.google.co.za/), Researchgate (https://www.researchgate.net/) and Academia (https://www.academia.edu/) to determine whether an archived copy existed. Unpaywall (https://unpaywall.org/) and the Open Access Button (https://openaccessbutton.org/) browser plugins were also used for this purpose. We did not include a search of any of the shadow libraries (Libgen or Sci-Hub).^7^ We accepted both published copies and archived post-prints (the post-print is the author’s version of an accepted article).

**Population Health Research Capsule**

What do we already know about this issue?

*Access to published research is limited for those without academic library access. This disproportionately affects less developed settings*.

What was the research question?

How accessible are the 500 most-cited emergency medicine articles?

What was the major finding of the study?

*Around 20% of publications were not accessible. Cost of access was significantly prohibitive. This limits global dissemination of knowledge*.

How does this improve population health?

*Publishing open access improves dissemination of knowledge, especially for those struggling with access in less developed settings*.

For articles that were still inaccessible, the corresponding authors were contacted (using their published emails, ResearchGate or Open Access Button) and asked to provide a copy of his or her article for a university research project. Corresponding authors were given 14 days to reply and were provided with full details of the study aims if they were requested. We collected article processing and access costs from each respective journal’s publisher. Publishers were contacted via email where cost information was not available on their public website.

We used the World Bank’s purchasing power parity (PPP) index to calculate the relative journal article processing and access cost differences for selected countries. PPP is based on the hypothesis that similar items cost the same no matter where in the world it is purchased. For instance, a tall Starbucks caffé latte will not just cost $2.95 in the United States (U.S.), but anywhere in the world; the only difference would be the expression of $2.95 in a foreign currency (R40.90 in South Africa). In reality, however, parity doesn’t exist. The PPP index describes this deviation from parity and uses the U.S. dollars as its baseline. A tall Starbucks caffé latte in South Africa actually costs R27.00 ($1.95) and not R40.90 ($2.95). For an American tourist ordering a tall Starbucks caffé latte in South Africa, this will result in a 33% cost saving, but for a South African tourist in the U.S. this will result in a 50% cost increase. Although not directly applicable to publication cost, the PPP index offers a simplified, hypothetical comparison of the relative article processing and access cost between countries, as it does for other goods. For our analysis we included only the top publishing countries of each global publication region, as per Scopus (North America, Europe, Middle East, Asia, South America, Africa and Pacific region).^8^ The top publishing country for each region were identified as the country with the largest EM publication output (number of articles) as described by SciVal. These were the U.S., Germany, Turkey, China, Brazil, South Africa, and Australia.

We employed Microsoft Excel (Redmond, Washington) for data analysis. Article accessibility was presented descriptively. The PPP index was used to calculate the factor by which publication costs differed between the included publishing countries, relative to the U.S. dollar. These were compared using a paired t-test, with significance defined as a p-value of less than 0.05. To provide an understanding of the economic burden of scientific publishing for scientists and clinicians living in middle-income countries, we calculated an equivalent local cost of article processing and access for the four middle-income countries included (South Africa, China, Turkey, and Brazil) to the U.S. cost of publishing and access, by applying the PPP index in reverse. Essentially this calculation allowed us to describe a similar out-of-pocket expense for a researcher earning in one of these four countries and the U.S.

The study protocol was reviewed and approved by both the Human Research Ethics committees of Stellenbosch University and the University of Cape Town, Cape Town, South Africa (largely due to involvement of an undergraduate researcher in the project).

## RESULTS

We collected the 501 top-ranked EM articles by citation count. After excluding one article due to its retraction from circulation, we were left with 500 articles published over 29 journals. Of these journals, 22 (76%) were hybrid open access journals (i.e., publish both open access articles and paid access articles), six (21%) were open access-only journals, and one was a subscription-only journal. One journal, *Critical Care and Resuscitation*, levies no article processing cost for open access publishing. However, as a society journal, access is restricted to members of the society for the first three months following publication, after which it is made universally accessible. There were 471 (94.2%) articles with first authors from high-income countries and 25 (5%) from upper-middle income countries, with the remaining four (0.8%), split equally between articles with first authors from lower-middle and lower-income countries.

**Figure 1:**
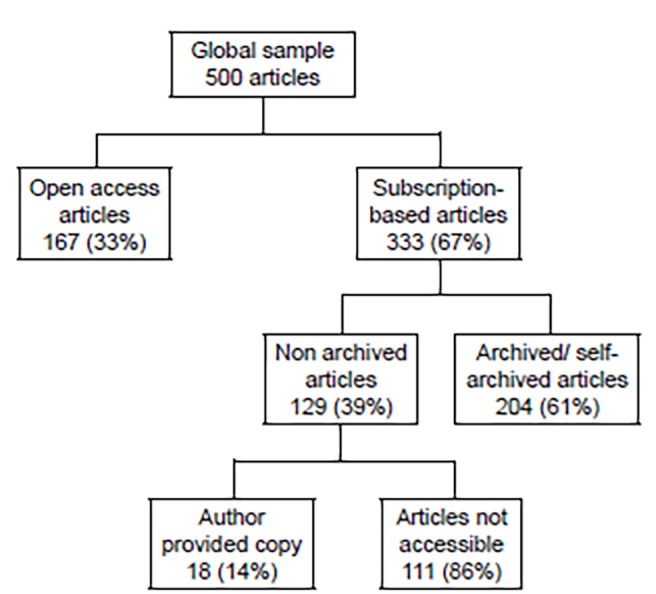
Flowchart describing access to the 500 most-cited emergency medicine articles between 2012–2016.

**Figure 2:**
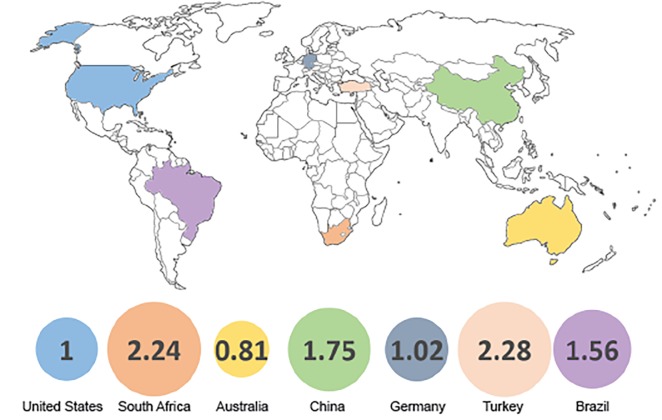
The factor by which publication costs differed between top publishing countries from each Scopus publishing region relative to the United States.

Figure 1 describes access to the top-cited 500 articles in EM. Of those articles, 111 (22%) were ultimately inaccessible without subscription. We excluded four journals from cost calculations as we were unable to locate any cost information on either the publisher’s website or on enquiry from the publisher. Figure 2 provides the factor by which published costs differed between the top publishing countries from each publishing region. A higher value implies a higher relative cost. The relative cost difference between the U.S. and the top publishing countries from other publishing regions was significant (p<0.001) for all countries except Australia (p=0.15) and Germany (p=0.27). The table provides equitable processing and access costs for the four low- and middle-income countries included in our sample (South Africa, China, Turkey, and Brazil), if the PPP index was applied in reverse to the mean U.S. article process and access costs.

## DISCUSSION

While two out of three of the top 500 cited EM articles were subscription based, only one in five were eventually found to need subscription for access. This figure broadly compares with the global open access rate, which is estimated at around 28% of peer-reviewed articles;^9^ however, little research exists on access to EM articles. One paper describes access to African EM articles, and shows much better access than described in our study: two-thirds of articles were accessible without subscription.^1^ This might be explained by the fact that authors from low- and middle-income countries can often apply for article processing cost waivers or discounts. However, authors from low-ranked institutions (which disproportionately occur in low- and middle-income countries) are less likely to publish open access despite such discounts.^3^

The cost of access to non-open access articles was significantly prohibitive for the low- and middle-income countries included in our sample. It is notable that waivers and discounts would not apply to any of these countries, as they are specifically excluded by publishers due to their *upper*-middle income status.^11^ For the same reason, these countries would not have access to the Research-for-life/Hinari Programme (a World Health Organisation initiative that provides free access to research for the poorest countries).^10^ It is worth noting that upper-middle income countries make up about 34% of the global population.^12^ It is likely that this aspect of access contributed to the creation of the shadow library SciHub, which also originated in an upper-middle income country. (A shadow library provides access to copyrighted books and research without the permission of authors and publishers.)^7^,^13^

Upper-middle income countries aside, the African EM open-access study showed that the relative cost of access was much higher for low- and lower-middle income countries. Relative to the U.S., costs were 3.5 and 2.8 times more for Ghanaian and Tanzanian authors, respectively.^1^ One explanation for this is that publication costs are driven by the supply and demand generated by the larger publication volumes in high-income countries. As a result, authors from low- and middle-income countries are forced to pay high-income country rates. This is likely to affect publication volume and subsequently knowledge dissemination in low- and middle-income countries.^9^

Apart from the social responsibility, publishing open access presents authors from high-income countries with an evidence-based opportunity to improve their citation counts (which is important for promotion and grant applications). Studies have shown that publishing open access improves discovery and citation of articles, offering a significant advantage.^14^

Although applications like Unpaywall and Open Access Button make it easier to find archived publications, it is more complicated than locating an open access article directly through its publisher’s website. Archiving is also dependent on publisher regulations, which often prohibit archiving for up to 12 months, and restricts which versions of an article can be archived.^3^ As archiving is not an automated process, authors also have to upload their own work manually.

Author responses to publication requests were less than half of what was observed in the African open access study. As the two cohorts differed substantially we did not explore this finding further. It is possible that the philanthropic nature of African authors played a role.

## LIMITATIONS

There are a number of limitations to this study. It is likely that many, or all, of these papers would be accessible through the shadow library SciHub. Publishers are clear that SciHub’s business model contravenes copyright. However, research has shown that scientists are often willing to view SciHub use in less black-and-white terms.^7^ Whatever the reader’s opinion might be, SciHub is likely to represent a symptom of a system that many feel is unjust and in need of change. Our study only considered a snapshot of the cost of access. Specifically we only considered the top publishing countries per Scopus publication region. Countries with weaker economies will likely face a much higher local cost for publication and access. Further studies can provide clarity regarding the relative cost differences. It is important to note that the PPP index reflects a relative difference for a basket of goods that does not include publication costs. Real market value would be determined by supply and demand, which will differ between goods even within the same economy. As a specific parity index for publishing costs does not exist, we used the PPP index for our calculations.

Our study only included articles from the Scopus database. A different database might have altered the findings of the study. However, Scopus does provide the largest abstract and citation database of peer-reviewed literature (including EM) globally, which explains our choice to use it. Finally, we only included articles from EM journals, which limits the list of top papers. Many top emergency care papers are published in leading non-EM journals with different access policies. This may also have affected the findings.

## CONCLUSION

In conclusion, this study showed that one in every five of the top 500 EM papers published in EM journals over a five-year period were not accessible without a subscription, and that access for scientists from low- and middle-income countries is significantly hampered by cost. It would be useful to view the uptake of open access over time to see if it is improving, as is happening in other specialties. Describing EM journals in terms of their accessibility (cost, self-archiving policies, etc.) and then linking this to journal impact might help guide authors select more accessible journals. Authors, specifically those from high-income countries, should consider the citation advantage afforded by open access publishing when deciding where to publish. It is also important to acknowledge the value this has for authors from low- and middle-income countries.

## Figures and Tables

**Table 1:** Equitable processing and access costs for four low- and middle-income countries if the purchasing power parity index was applied in reverse to mean U.S. article processing and access costs.

Cost variable	Mean cost (U.S.)	South Africa	China	Turkey	Brazil
Processing	$2,518.62	$1,125.75	$1,441.30	$1,102.50	$1,613.26
Single paper access	$44.78	$20.02	$25.63	$19.60	$28.68

*U.S*., United States.
